# Vitamin D supplementation as an adjuvant therapy for patients with T2DM: an 18-month prospective interventional study

**DOI:** 10.1186/1475-2840-11-85

**Published:** 2012-07-18

**Authors:** Nasser M Al-Daghri, Khalid M Alkharfy, Abdulaziz Al-Othman, Emad El-Kholie, Osama Moharram, Majed S Alokail, Yousef Al-Saleh, Shaun Sabico, Sudhesh Kumar, George P Chrousos

**Affiliations:** 1 Biomarkers Research Program, Biochemistry Department, College of Science, King Saud University, PO Box, 2455, Riyadh, 11451, Kingdom of Saudi Arabia; 2 Center of Excellence in Biotechnology Research Center, King Saud University, Riyadh, 11451, Kingdom of Saudi Arabia; 3 Prince Mutaib Chair for Biomarkers of Osteoporosis, King Saud University, Riyadh, 11451, Kingdom of Saudi Arabia; 4 Clinical Pharmacy Department, College of Pharmacy, King Saud University, Riyadh, 11451, Kingdom of Saudi Arabia; 5 College of Applied Medical Sciences, King Saud University, Riyadh, 11451, Kingdom of Saudi Arabia; 6 King Abdulaziz University Hospital, King Saud University, Riyadh, 11451, Kingdom of Saudi Arabia; 7 College of Medicine, King Saud University for Health Sciences, Riyadh, 11426, Kingdom of Saudi Arabia; 8 Clinical Sciences Research Institute, Diabetes and Metabolism Unit, Coventry, CV47AL, UK; 9 First Department of Pediatrics, Athens University Medical School, Athens, 11527, Greece

**Keywords:** Vitamin D, Diabetes mellitus, Saudi, Supplementation

## Abstract

**Background:**

Vitamin D deficiency has been associated with impaired human insulin action, suggesting a role in the pathogenesis of diabetes mellitus type 2 (T2DM). In this prospective interventional study we investigated the effects of vitamin D3 supplementation on the metabolic profiles of Saudi T2DM subjects pre- and post-vitamin D supplementation over an 18-month period.

**Methods:**

T2DM Saudi subjects (men, N = 34: Age: 56.6 ± 8.7 yr, BMI, 29.1 ± 3.3 kg/m^2^; women, N = 58: Age: 51.2 ± 10.6 yr, BMI 34.3 ± 4.9 kg/m^2^;) were recruited and given 2000 IU vitamin D3 daily for 18 months. Anthropometrics and fasting blood were collected (0, 6, 12, 18 months) to monitor serum 25-hydroxyvitamin D using specific ELISA, and to determine metabolic profiles by standard methods.

**Results:**

In all subjects there was a significant increase in mean 25-hydroxyvitamin D levels from baseline (32.2 ± 1.5 nmol/L) to 18 months (54.7 ± 1.5 nmol/L; * p * < 0.001), as well as serum calcium (baseline = 2.3 ± 0.23 mmol/L vs. 18 months = 2.6 ± 0.1 mmol/L; *p* = 0.003). A significant decrease in LDL- (baseline = 4.4 ± 0.8 mmol/L vs. 18 months = 3.6 ± 0.8 mmol/L,* p * < 0.001] and total cholesterol (baseline = 5.4 ± 0.2 mmol/L vs. 18 months = 4.9 ± 0.3 mmol/L, *p* < 0.001) were noted, as well as a significant improvement in HOMA-β function (* p * = 0.002). Majority of the improvements elicited were more prominent in women than men.

**Conclusion:**

In the Saudi T2DM population receiving oral Vitamin D3 supplementation (2000 IU/day), circulating 25-hydroxyvitamin D levels remained below normal 18 months after the onset of treatment. Yet, this “suboptimal” supplementation significantly improved lipid profile with a favorable change in HDL/LDL ratio, and HOMA-β function, which were more pronounced in T2DM females.

## Background

In recent years, vitamin D deficiency has gained unprecedented attention in the fields of preventive cardiology and endocrinology, primarily because of the extra-skeletal pleiotropic effects of this hormone, and the association of its deficit with insulin resistance, diabetes mellitus and an increased cardiovascular risk [1-3]. A unifying factor that links diabetes mellitus types 1 and 2 (T1DM and T2DM, respectively) is the expression of vitamin D receptors (VDRs) in more than 30 biological tissues, including the pancreatic islet cells [3-5]. On the other hand, it is common for patients with T1DM and T2DM to have vitamin D deficiency [6]. Furthermore, several longitudinal and observational studies have demonstrated that low levels of serum 25-hydroxyvitamin D predict T2DM risk in Europeans [7,8], African-Americans [9], South Asians [10] and native American children [11]. Vitamin D correction, therefore, may increase insulin secretion and improve glucose homeostasis; however, its effects on healthy individuals or in those with impaired glucose tolerance remain unclear [6].

In the Middle East and North Africa (MENA), vitamin D deficiency is startlingly high as compared to other geographical regions [12]. Specifically, in the kingdom of Saudi Arabia (KSA), where the intensity of sunlight is paradoxically directly proportional to the prevalence of vitamin D deficiency [13], hypovitaminosis D has been implicated not only as a significant predictor of osteoporosis among apparently healthy men and post-menopausal women [14,15], but also significantly associated to cardiometabolic risk factors in both children and adults [16,17]. Interestingly, there is an equally alarming prevalence of chronic non-communicable diseases in KSA, including T2DM, obesity, the metabolic syndrome, and cardiovascular diseases [18].

Previously we demonstrated a modest reversal of metabolic syndrome manifestations among a cohort of adult Saudis who were advised vitamin D correction through increased sun exposure and dietary vitamin D intake [19]. In the current interventional study, we aimed to determine the metabolic effects of oral vitamin D supplementation in a cohort of T2DM Saudi subjects over time. This study may shed light as to whether oral vitamin D supplementation can be an adjunct therapy in T2DM Saudi patients, and on the importance of optimizing vitamin D levels in the prevention and improved management of chronic non-communicable diseases such as T2DM.

## Methods

### Site and duration of the study

This is a multi-center, interventional study conducted at the primary health care out -patient clinics in Riyadh, Kingdom of Saudi Arabia. Ethics approval was obtained from the ethics committee of the College of Medicine Research Center at King Saud University, Riyadh, KSA.

### Subjects

A total of 120 adult Saudi patients (males and females) with controlled T2DM (known cases), aged 30 year and above but not exceeding 70 years, were randomly recruited for the study. Subjects were excluded if taking mineral oil products, using antacids regularly, taking cortisone or other steroids, diuretics, weight-loss drugs, phenobarbital and phenytoin, having liver problems, gallbladder disease or gastrointestinal disorders and taking daily multivitamins. Patients receiving hypoglycemic agents and statins were allowed in the study and changes in drug doses were noted.

All subjects were given a general questionnaire, which includes thorough past and present medical history. They then underwent a physical examination and required to submit written informed consents prior to being included in the study. All subjects were given a six-month supply of 2000 IU Vitamin D3 tablets (Vigantoletten; Merck Pharma, Germany) to be taken daily, after which and every 6 months they were to return to their assigned primary care center for another examination and prescription refill.

### Anthropometry

All anthropometric parameters were obtained while the subject was standing erect and barefoot. Height and weight were determined using standardized conventional methods. Body mass index (BMI) was calculated using the formula: weight in kilograms (kg) divided by height in squared meters (m²). A standardized mercurial sphygmomanometer was used to take the blood pressure of each participant 30 min after complete rest.

### Laboratory parameters

Fasting blood samples were collected at baseline for quantification of different metabolic parameters. This procedure was repeated every 6 months prior to the refill of the next vitamin D supplements. Fasting blood glucose, lipid profile, albumin, phosphorous and calcium were determined using routine laboratory procedures (Konelab, Espoo, Finland). Serum insulin was quantified using multiplex assay kits (Luminex® xMAP® Technology platform) (Luminexcorp, Texas). The intra-assay variation was 1.4–7.9% and inter-assay variation of <21%. Minimum detectable concentration (MDC) for insulin was 50.9 pg/ml. Parathyroid hormone (PTH) and 25-hydroxy-vitamin D were measured using enzyme-linked immunosorbent assay (IDS Ltd., Boldon Colliery, Tyne & Wear, UK). The inter- and intra-assay variabilities were 5.8% and 3.4%, for intact PTH, and 5.3% and 4.6%, for 25-hydroxyvitamin D, respectively. All measurements were performed in a DEQA- (Vitamin D External Quality Assessment) participating laboratory, the Biomarkers Research Program (BRP) of King Saud University, Riyadh, KSA.

Homeostasis model assessment for insulin resistance (HOMA-IR) was calculated as fasting insulin (IU) x fasting glucose (mmol/L)/22.5. HOMA-β secretion (%) was calculated as 20 x fasting insulin (IU)/(fasting glucose—3.5) [20]. Vitamin D deficiency was defined as serum 25-hydroxyvitamin D level <50 nmol/L.

### Statistical analysis

The Statistical Package for the Social Sciences (SSPS) for Windows version 16.5 (Chicago, Illinois) was used for statistical evaluation of data. Variables exhibiting non-Gaussian distribution were logarithmically transformed. Repeated measures analysis of variance (ANOVA) was undertaken to compare values over time. A p-value < 0.05 was deemed significant.

## Results

### Improved vitamin D and metabolic profile after 18 months of supplementation

The general characteristics of all T2DM subjects are presented in Table 1. Vitamin D deficiency was noted in all subjects. Despite supplementation, all subjects remained vitamin D deficient by as much as 22% even after 18 months of therapy. None of the subjects reported changes in their oral hypoglycemic agent, insulin and statin dosage. Of the 120 subjects who enrolled, 92 subjects (34 males and 58 females) (23.3% attrition rate) were able to complete the entirety of the 18 month supplementation. There was a significant improvement in the circulating levels of 25-hydroxyvitamin D from baseline to 6 months (32.2 ± 1.5 vs. 57.7 ± 1.4 nmol/l) (p < 0.001), and these levels remained unchanged over the course of the supplementation period. In parallel, a significant improvement in the lipid profile of subjects was observed as evidenced by a decrease in total cholesterol (4.8 ± 0.28 mmol/l) as compared to baseline (5.4 ± 0.21 mmol/l) (p < 0.001), as well as LDL-cholesterol (4.3 ± 0.93 vs. 3.7 ± 1.0 mmol/l) (p = 0.004) after 18 months. Worthy to note was the non-significant increase in HDL-cholesterol across time points. Other metabolic parameters that changed significantly were an increase of serum calcium (p = 0.003); insulin (p < 0.001) and HOMA-IR (p < 0.001), and HOMA-β cell function (p = 0.002). Of note was the significant decrease in serum albumin (p < 0.001). The rest of the parameters (blood pressure, BMI, glucose, serum magnesium and phosphorus were similar.

**Table 1:** General characteristics of all T2DM subjects according to follow-up

	**Baseline**	**6 Months**	**12 Months**	**18 Months**	**P value**
N	92				
M/F	34/58				
DMT2 Duration (years)	7.2 ± 5.9				
Insulin (N%)	18 (19.6)				
Sulfonylurea (N%)	68 (73.9)				
Statins (N%)	44 (47.8)				
Diet (N%)	6 (6.5)				
Age (years)	53.9 ± 10.2				
Systolic BP (mmHg)	126.0 ± 14.1	125.3 ± 17.2	128.4 ± 14.4	126.1 ± 8.1	0.46
Diastolic BP (mmHg)	79.6 ± 7.3	79.0 ± 10.1	79.6 ± 9.5	77.2 ± 6.2	0.27
BMI (kg/m^2^)	32.5 ± 5.0	32.6 ± 4.9	32.8 ± 4.9	33.2 ± 5.2	0.12
Glucose (mmol/l)	10.7 ± 3.8	11.0 ± 4.3	11.7 ± 4.1	10.9 ± 4.4	0.21
T. Cholesterol (mmol/l)	5.4 ± 0.21	5.2 ± 0.25	4.9 ± 0.22*	4.8 ± 0.28*	<0.001
Triglycerides (mmol/l)	1.9 ± 0.33	2.0 ± 0.32	1.9 ± 0.26	1.9 ± 0.34	0.15
HDL-Chol (mmol/l)	1.0 ± 0.35	1.1 ± 0.26	1.12 ± 0.27	1.2 ± 0.37	0.38
LDL-Chol (mmol/l)	4.3 ± 0.93	4.0 ± 0.85	3.7 ± 1.1*	3.7 ± 1.0*	0.004
Corr. Calcium (mmol/l)	2.3 ± 0.23	2.4 ± 0.17	2.5 ± 0.22	2.56 ± 0.14*	0.003
Albumin (g/L)	43.5 ± 6.5	40.3 ± 6.6	40.0 ± 4.0*	39.5 ± 4.7*	<0.001
Insulin (IU/ml)	14.0 ± 2.0	18.9 ± 2.3	27.0 ± 1.8*	24.5 ± 2.1*	<0.001
PTH (pmole/l)	1.3 ± 0.39	0.96 ± 0.24	0.91 ± 0.22*	0.92 ± 0.23*	0.02
HOMA-IR	6.2 ± 1.0	8.0 ± 1.0	11.9 ± 1.0*	11.4 ± 1.4*	<0.001
HOMA β-cell function	52.1 ± 9.0	72.5 ± 15.3	82.7 ± 9.5*	96.5 ± 15.3*	0.002
25-(OH) D (nmol/l)	32.2 ± 1.5	57.7 ± 1.4*	48.1 ± 1.4*#	54.7 ± 1.5*	<0.001
Magnesium (mmol/l)	0.69 ± 0.11	0.67 ± 0.08	0.66 ± 0.05	0.70 ± 0.15	0.65
Phosphorus (mmol/l)	1.17 ± 0.23	1.17 ± 0.23	1.13 ± 0.22	1.11 ± 0.23	0.16

**Note**: Data presented as mean ± SD; * denotes significance compared to baseline; # denotes significance compared to 6 months; P-value significant at <0.05.

### Gender differences in metabolic improvements after vitamin D supplementation

**Figure 1:**
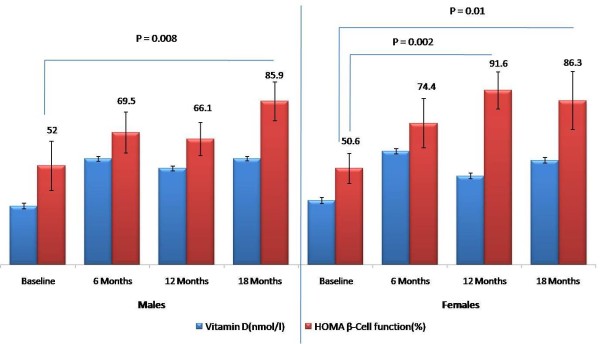
HOMA-β cell function (%) levels versus vitamin D (nmol/L) in male and female Saudi T2DM subjects who were on daily vitamin D3 (2000 IU) supplementation for 18 months in successive follow-up visits; significance at P < 0.05.

We examined whether there were apparent differences in the improvement of metabolic profile in males vs. females (Table 2). In males, there was an increase in the circulating levels of 25-hydroxyvitamin D that was apparent on the 6 month evaluation (30.9 ± 1.4 vs. 55.7 ± 1.3 nmol/l, p < 0.001). A significant increase was also observed in serum calcium levels in men but not women (p = 0.03). The improvement in metabolic profile was more apparent in females, and showing improved levels in total and LDL-cholesterol (p < 0.001 and 0.004, respectively), albumin (p < 0.001), insulin (p < 0.001), HOMA-IR (p < 0.01) and HOMA-β cell function (p < 0.004), as well as 25-hydroxyvitamin D (p < 0.001). Figure 1 shows the increasing pattern of HOMA-β secretion improvement over time despite a sustained, almost significant increase in vitamin D levels beyond 6 months.

**Table 2:** General characteristics of male and female T2DM subjects according to follow-up

	**MALES**					**FEMALES**				
	**Baseline**	**6 Months**	**12 Months**	**18 Months**	**P val**	**Baseline**	**6 Months**	**12 Months**	**18 Months**	**P val**
N	34					58				
DMT2 Duration (years)	7.3 ± 6.5					7.1 ± 5.5				
Age (years)	56.6 ± 8.7					51.2 ± 10.6				
Systolic BP (mmHg)	127.0 ± 15.2	125.2 ± 17.5	131.7 ± 16.2	124.9 ± 8.5	0.05	127.3 ± 13.4	125.4 ± 17.2	126.3 ± 12.9	126.7 ± 7.8	0.90
Diastolic BP (mmHg)	79.3 ± 7.4	80.4 ± 10.1	80.7 ± 8.5	76.2 ± 5.1	0.10	79.7 ± 7.3	78.0 ± 10.1	78.9 ± 10.1	77.9 ± 7.1	0.72
BMI (kg/m^2^)	29.0 ± 3.3	29.2 ± 3.7	29.1 ± 3.7	29.7 ± 4.3	0.41	34.2 ± 4.9	34.4 ± 4.6	34.7 ± 4.5	3.4.9 ± 4.7	0.30
Glucose (mmol/l)	10.9 ± 4.6	10.8 ± 3.8	12.8 ± 3.9	11.3 ± 5.2	0.11	10.6 ± 3.4	11.2 ± 4.6	11.2 ± 4.1	10.8 ± 3.9	0.70
T Cholesterol (mmol/l)	5.4 ± 1.2	5.5 ± 1.6	5.0 ± 1.0	4.9 ± 1.1	0.11	5.5 ± 0.84	5.3 ± 1.0	4.9 ± 0.99*	4.9 ± 1.2*	<0.001
Triglycerides (mmol/l)	1.8 ± 0.31	2.1 ± 0.32	2.0 ± 0.29	1.8 ± 0.32	0.25	1.9 ± 0.35	2.0 ± 0.33	1.7 ± 0.25	1.9 ± 0.34	0.14
HDL-Chol (mmol/l)	1.4 ± 0.29	1.1 ± 0.07	1.1 ± 0.11	1.1 ± 0.12	0.10	1.0 ± 0.12	1.1 ± 0.13	1.1 ± 0.13	1.2 ± 0.17	0.16
LDL-Chol (mmol/l)	4.2 ± 1.5	4.1 ± 1.4	4.0 ± 1.9	4.1 ± 2.4	0.68	4.4 ± 0.80	4.0 ± 0.77	3.6 ± 0.90*	3.6 ± 0.77*	0.004
Corr. Calcium (mmol/l)	2.2 ± 0.22	2.4 ± 0.19	2.5 ± 0.27*	2.5 ± 0.10*	0.03	2.4 ± 0.21	2.4 ± 0.16	2.5 ± 0.19	2.5 ± 0.17	0.06
Albumin (g/L)	47.0 ± 7.9	43.9 ± 9.0	42.3 ± 3.0	42.6 ± 4.4	0.11	41.6 ± 4.5	38.3 ± 3.4*	39.4 ± 3.9*	37.7 ± 4.0*	<0.001
Insulin (IU/ml)	15.2 ± 1.9	18.9 ± 2.2	24.5 ± 2.1	21.5 ± 2.1	0.23	13.0 ± 2.1	19.0 ± 2.4	28.5 ± 1.6*	22.0 ± 2.2*	0.001
PTH (pmole/l)	1.1 ± 0.32	0.88 ± 0.20	0.89 ± 0.23	0.91 ± 0.24	0.06	1.4 ± 0.44	0.99 ± 0.34	0.97 ± 0.35	0.98 ± 0.32	0.09
HOMA-IR	6.8 ± 0.86	8.2 ± 0.96	11.1 ± 0.99	12.2 ± 1.1	0.32	5.8 ± 1.0	8.2 ± 0.99	12.4 ± 0.97*	11.2 ± 1.3*	0.01
HOMA β function (%)	52.0 ± 12.9	69.5 ± 10.7	66.1 ± 8.7	85.9 ± 15.3	0.12	50.6 ± 7.8	74.4 ± 18.1	91.6 ± 9.8*	86.3 ± 15.2*	0.004
25-(OH) D (nmol/l)	30.9 ± 1.4	55.7 ± 1.3*	50.6 ± 1.3*	55.7 ± 1.2*	<0.001	33.7 ± 1.6	59.7 ± 1.4*	46.7 ± 1.5*	54.9 ± 1.5*	<0.001
Magnesium (mmol/l)	0.71 ± 0.19	0.7 ± 0.07	0.70 ± 0.04	0.71 ± 0.05	0.90	0.69 ± 0.08	0.65 ± 0.08	0.65 ± 0.04	0.70 ± 0.19	0.73
Phosphorus (mmol/l)	1.20 ± 0.25	1.15 ± 0.21	1.05 ± 0.22	1.04 ± 0.19*	0.009	1.15 ± 0.22	1.20 ± 0.21	1.17 ± 0.22	1.16 ± 0.25	0.52

**Note**: Data presented as mean ± SD; * denotes significance compared to baseline; # denotes significance compared to 6 months; P-value significant at <0.05.

## Discussion

The findings of this study indicate that the metabolic profile of T2DM subjects, particularly females, is significantly improved over a period of 18 months after the onset of vitamin D3 supplementation, suggesting that vitamin D correction is a promising cardio-protective intervention in vitamin D-deficient populations. The gender effect can be attributed indirectly to either differences in hormone secretion and/or target tissue effects and body fat distribution, both of which can contribute to degrees of insulin resistance, or sample size effect. The current study adds to an increasing body of evidence that vitamin D supplementation is most beneficial not only to those who are at risk for osteoporosis and other bone-related diseases, but also to those who are deficient and have other extra skeletal chronic diseases, such as diabetes T2DM and cardiovascular disease. The available evidence, however, has been quite mixed. In a recent study, Eftekhari and colleagues were not able to elicit the same improved metabolic profile in an Iranian T2DM population, which was probably due to a shorter duration of supplementation (12 weeks) [21]. Other studies also found no improvement of insulin sensitivity after a high dose vitamin D intervention. This was probably partly due to the supplementation itself and/or because the subjects were apparently healthy [22,23]. Increased insulin resistance post supplementation was observed in a cohort of middle-aged adults, and increased insulin sensitivity in first time GDM patients [24,25]. In the present study, the apparent health benefits were observed after 6 months of supplementation, aside from the expected increase in circulating vitamin D levels, suggesting that sustained and prolonged supplementation might be necessary to achieve desirable metabolic effects.

Several mechanisms explain how vitamin D can theoretically improve metabolic functions. One mechanism may involve direct promotion of large HDL particle formation, via elevations in serum apolipoprotein A1 (ApoA1) concentrations, a process that increases reverse cholesterol transport [26]. Furthermore, the improved lipid profile of the DMT2 subjects can also be attributed to lipid-lowering drugs, which were administered in almost half of the study population. Statins (rosuvastatin) were shown to increase levels of 25-hydroxyvitamin D and 1, 25 dihydroxyvitamin D in a cohort of hyperlipidemic patients [27]. Nevertheless, there is enough evidence to support that vitamin D supplementation can independently improve cardiovascular health secondary to its significant associations with cardiometabolic risk factors in both human and animal models, including blood pressure, insulin resistance and aortic media fragmentation, respectively [28-30]. The increase in serum calcium was expected, secondary to increased vitamin D levels. This in turn results in a parallel increase in localized calcium influx in pancreatic β cells that stimulate islet insulin secretion [31-33]. Lastly, the inverse association to free albumin was also expected, as it is one of the proteins (vitamin D binding protein being the other one) that transport 25(OH)D in the blood [34].

Several caveats should be mentioned. First, the lack of a placebo-control group, second that the study included only patients with T2DM, and third, the difficulty in the verification of quality of life issues. Findings therefore are limited to patients with T2DM, since variations in metabolic changes differ not only by gender but also by the presence of the disease itself [35]. The increase in both insulin resistance and sensitivity should be interpreted with caution since these were computed indirectly rather than directly. Nevertheless, several hard outcomes were measured overtime, while HOMA-β function would be difficult to elicit in a control group. The improved HOMA-β function specifically in females can be also explained by several confounders that were not accounted for, such as the type of anti-diabetic drugs taken, compliance of the patients and duration of diabetes. Metformin for instance, does not confer changes in vitamin D status, while thiazolidinediones affect vitamin D by selective agonism of peroxisome proliferator activated receptors gamma (PPAR-γ), which are present in muscle, liver and adipose tissue [36].

In summary, the present interventional study performed in an Arab population suggests that daily 2000 IU vitamin D3 supplementation in a vitamin D deficient T2DM population is associated with measurable cardioprotective indices. Supplementation to achieve higher levels of vitamin D remains a promising adjuvant therapy for T2DM patients. Further studies are needed, with the inclusion of a placebo group, to validate the present findings.

## Competing interests

The authors have no conflict of interest to disclose related to this study.

## Authors’ contributions

NMA and KMA conceived the study. AA, EE and OM recruited subjects and collected data. MSA and YA recruited subjects analyzed samples. SS performed data analysis and wrote the manuscript. SK and GC reviewed/edited the final version of the manuscript. All authors approved the final manuscript.
